# Therapeutic efficacy of zolpidem combined with cognitive-behavioral therapy on primary insomnia

**DOI:** 10.1097/MD.0000000000017122

**Published:** 2019-09-27

**Authors:** Ying Song, Bing Liang

**Affiliations:** a Department of Neurology, The Fifth Central Hospital of Tianjin Binhai Hospital of Peking University; b Department of Characteristic Medical Center, Chinese People's Armed Police Force, Tianjin, China.

**Keywords:** cognitive-behavioral therapy, efficacy, primary insomnia, randomized controlled trial, safety, zolpidem

## Abstract

**Background:**

In this study, we intend to assess the efficacy of zolpidem combined with cognitive-behavioral therapy (CBT) for patients with primary insomnia (PI).

**Methods:**

A predefined search strategy will be used to search for associated literature from inception to the July 1, 2019: PubMed, EMBASE, Cochrane Library, Scopus, Web of Science, Google Scholar, Chinese Biomedical Literature Database, and China National Knowledge Infrastructure with no language limitation. In addition, we will also retrieve reference lists of included studies and relevant reviews, as well as the conference proceedings. All randomized controlled trials related to the zolpidem and CBT for PI will be included. Two authors will perform study selection, data collection, and study quality, respectively. We will also apply RevMan 5.3 software for statistical analysis.

**Results:**

This study will provide a comprehensive overview of the available evidence of the benefits and safety of zolpidem and CBT for PI. Primary outcomes are sleep quality and severity of sleep disorders. Secondary outcomes consist of sleep-onset latency, total sleep duration, sleep efficiency, and frequency and adverse events.

**Conclusion:**

The results of this study will inform clinical and policy decisions regarding the benefits and harm of zolpidem and CBT for patients with PI.

**PROSPERO registration number:**

PROSPERO CRD42019142796.

## 1 Introduction

Primary insomnia (PI) is a very serious sleep disturbance.^[1,2]^ This disorder comprises of acute, subchronic and persistent insomnia according to the duration of PI.^[3–6]^ Such condition is often secondary to the multiple factors, including headache, anxiety, depression, cardio-cerebrovascular diseases, or psychiatric issues.^[8–13]^ It has been estimated that about 10% to 20% of the population worldwide suffer from poor sleep quality,^[14]^ and approximately 50% of those population experience more than 1 month.^[14]^ The incidence of PI is more likely to affect female than male individuals.^[15,16]^

A variety of managements can be used to treat PI, such as eszopiclone, doxepin, acupuncture, physical exercise, zolpidem, and cognitive-behavioral therapy (CBT).^[17–24]^ However, there is still limited efficacy of those single therapies. Thus, it is very important to apply combined treatments for the treatment of patients with PI, such as combination of zolpidem and CBT.^[25–28]^ However, the efficacy is still inconclusive. Therefore, this study aims to assess the efficacy and safety of zolpidem and CBT for the treatment of patients with PI systematically.

## 2 Methods

### 2.1 Inclusion criteria for study selection

#### 2.1.1 Study types

All randomized controlled trials (RCTs) of zolpidem and CBT for the management of PI will be included without language or publication status limitation.

#### 2.1.2 Participant types

All participants with diagnosed PI will be included without limitation of gender, age, and ethnic background.

#### 2.1.3 Intervention types

The therapy used in the experimental group includes combination of zolpidem and CBT.

The control group can be any interventions, except any forms of zolpidem and CBT.

#### 2.1.4 Outcome types

Primary outcomes are sleep quality and severity of sleep disorders, as assessed by Pittsburgh sleep quality index or other relevant scales.

Secondary outcomes consist of sleep-onset latency, total sleep duration, sleep efficiency, and frequency and adverse events.

### 2.2 Data sources and search methods

Eight databases of PubMed, EMBASE, Cochrane Library, Scopus, Web of Science, Google Scholar, Chinese Biomedical Literature Database, and China National Knowledge Infrastructure will be comprehensively searched from inception to the July 1, 2019 with no language limitation for the RCTs regarding zolpidem and CBT for PI. The detailed strategy for PubMed is presented in Table 1. Any modified search strategies will be applied for other electronic databases. Relevant conference proceedings, reference list of eligible studies, and relevant reviews will also be searched.

**Table 1:**
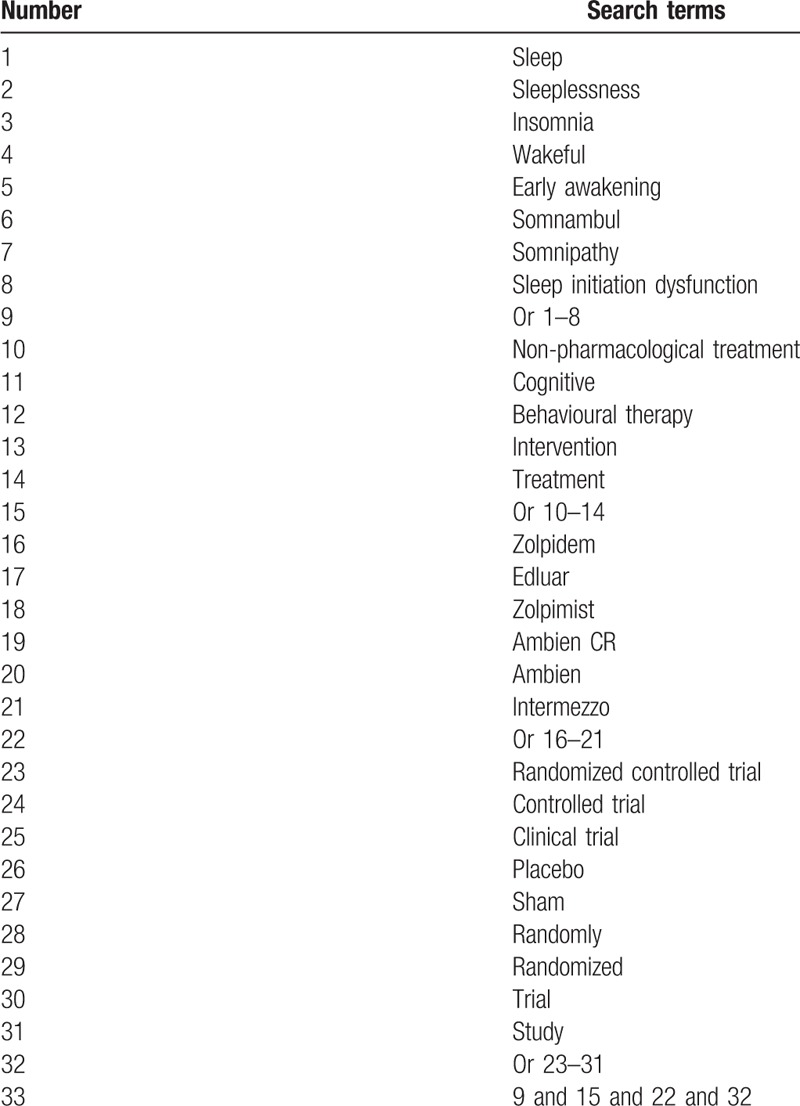
Search strategy of PubMed database.

### 2.3 Data collection

#### 2.3.1 Study selection

Two authors will independently carry out study selection according to the previous designed eligibility criteria. All literature records will be scanned and all obvious disqualified studies will be excluded through the titles and abstracts. The final full text will be read to judge whether they meet all inclusion criteria. Any inconsistencies between 2 authors will be solved by another author via discussion. The process of study selection will be presented in the flowchart.

#### 2.3.2 Data extraction

Two authors will independently extract the data via a standardized data form. Any disagreements will be checked and arbitrated by a third author through discussion. This sheet includes basic general information (authors, titles, year of publication, age, etc), disease duration, study setting, study methods, sample size, treatment details, comparators, outcome details, adverse events, and conflicts of interest.

#### 2.3.3 Dealing with missing data

We will correspond with the primary author to inquire the missing or insufficient or unclear data. If we can not obtain those data, only available data will be analyzed and its potential impact will be discussed.

### 2.4 Assessment of risk of bias

Two authors will independently assess the risk of bias using the Cochrane Risk of Bias Tool. Any discrepancies between 2 authors will be solved by a third author via discussion. This tool consists of 7 aspects and each one will be categorized into 3 levels of high, unclear, or low risk of bias.

### 2.5 Measures of treatment effect

For continuous data, a standard mean difference and 95% confidence interval will for calculation. For dichotomous outcomes, a rate ratio and 95% confidence interval will be expressed for treatment effect measurement.

### 2.6 Assessment of heterogeneity

We will use *I*^*2*^ test to identify heterogeneity among included studies. If a value of *I*^*2*^ less than 50%, it will be regarded as acceptable. Otherwise, if an *I*^*2*^ value exceeds 50%, it will be considered as substantial. At the same time, subgroup analysis will be performed to explore the potential causes of heterogeneity.

### 2.7 Data synthesis and analysis

RevMan 5.3 software will be employed to compute the data pooling when a meta-analysis is provided. If *I*^*2*^ ≤50%, a fixed-effects model will be used for data pooling. If *I*^*2*^ >50%, a random-effects model will be used to perform data pooling, and subgroup analysis will be conducted. If data are limited or significant heterogeneous to pool after subgroup analysis, we will summarize findings in a narrative review.

### 2.8 Subgroup analysis

According to the different treatments, controls, and outcomes, subgroup analysis will be carried out to explore the resources of heterogeneity if eligible studies are sufficient.

### 2.9 Sensitivity analysis

We will conduct sensitivity analysis to identify the robustness of outcome results by removing low-quality studies.

### 2.10 Reporting bias

We will perform the Funnel plot^[29]^ and Egger regression test^[30]^ to identify any possible reporting bias if more than 10 trials entered.

## 3 Discussion

PI is one of the most frequency disorders among general population. A variety of managements are used for the treatment of PI. Zolpidem and CBT have been used in various clinical conditions, including PI. To the best of our knowledge, the efficacy and safety of zolpidem and CBT have not been clearly elucidated systematically yet. Therefore, it is very necessary to carry out a high-quality study systematically, and the process of this study will be presented in the diagram. It is expected that this study can provide rigorous and objective evidences of the efficacy and safety of zolpidem and CBT for patients with PI.

## Author contributions

**Conceptualization:** Ying Song, Bing Liang.

**Data curation:** Ying Song, Bing Liang.

**Formal analysis:** Bing Liang.

**Funding acquisition:** Ying Song.

**Investigation:** Ying Song.

**Methodology:** Bing Liang.

**Project administration:** Ying Song.

**Resources:** Bing Liang.

**Software:** Bing Liang.

**Supervision:** Ying Song.

**Validation:** Ying Song, Bing Liang.

**Visualization:** Ying Song, Bing Liang.

**Writing – original draft:** Ying Song, Bing Liang.

**Writing – review and editing:** Ying Song, Bing Liang.

## References

[1] Pigeon WR Perlis ML Sleep homeostasis in primary insomnia. Sleep Med Rev 2006;10:247–54. 16563817 10.1016/j.smrv.2005.09.002

[2] Cochran H Diagnose and treat primary insomnia. Nurse Pract 2003;28:13–27. 10.1097/00006205-200309000-00003 14501552

[3] Reynolds CF 3rd Kupfer DJ Buysse DJ Subtyping DSM-III-R primary insomnia: a literature review by the DSM-IV Work Group on Sleep Disorders. Am J Psychiatry 1991;148:432–8. 2006686 10.1176/ajp.148.4.432

[4] Gillam T Understanding primary insomnia in older people. Nurs Older People 2009;21:30–3. 10.7748/nop2009.04.21.3.30.c7014 19363949

[5] Edinger JD Krystal AD Subtyping primary insomnia: is sleep state misperception a distinct clinical entity? Sleep Med Rev 2003;7:203–14. 12927120 10.1053/smrv.2002.0253

[6] Perlis ML Youngstedt SD The diagnosis of primary insomnia and treatment alternatives. Compr Ther 2000;26:298–306. 11126102 10.1007/s12019-000-0033-6

[7] Srinivasan V Brzezinski A Pandi-Perumal SR Melatonin agonists in primary insomnia and depression-associated insomnia: are they superior to sedative-hypnotics? Prog Neuropsychopharmacol Biol Psychiatry 2011;35:913–23. 21453740 10.1016/j.pnpbp.2011.03.013

[8] Tran DP Spierings EL Headache and insomnia: their relation reviewed. Cranio 2013;31:165–70. 23971156 10.1179/crn.2013.026

[9] Staner L Comorbidity of insomnia and depression. Sleep Med Rev 2010;14:35–46. 19939713 10.1016/j.smrv.2009.09.003

[10] Nowell PD Reynolds CF 3rd Buysse DJ Paroxetine in the treatment of primary insomnia: preliminary clinical and electroencephalogram sleep data. J Clin Psychiatry 1999;60:89–95. 10.4088/jcp.v60n0204 10084634

[11] Roth T Zammit G Lankford A Nonrestorative sleep as a distinct component of insomnia. Sleep 2010;33:449–58. 20394313 10.1093/sleep/33.4.449 PMC2849783

[12] Baglioni C Battagliese G Feige B Insomnia as a predictor of depression: a meta-analytic evaluation of longitudinal epidemiological studies. J Affect Disord 2011;135:10–9. 21300408 10.1016/j.jad.2011.01.011

[13] Jaussent I Empana JP Ancelin ML Insomnia, daytime sleepiness and cardio-cerebrovascular diseases in the elderly: a 6-year prospective study. PLoS One 2013;8:e56048. 23457496 10.1371/journal.pone.0056048 PMC3573087

[14] Buysse DJ Insomnia. JAMA 2013;309:706–16. 23423416 10.1001/jama.2013.193 PMC3632369

[15] Yoshioka E Saijo Y Kita T Gender differences in insomnia and the role of paid work and family responsibilities. Soc Psychiatry Psychiatr Epidemiol 2012;47:651–62. 21476013 10.1007/s00127-011-0370-z

[16] Hale L Do DP Basurto-Davila R Does mental health history explain gender disparities in insomnia symptoms among young adults? Sleep Med 2009;10:1118–23. 19467926 10.1016/j.sleep.2008.12.011 PMC2805081

[17] Scharf M Erman M Rosenberg R A 2-week efficacy and safety study of eszopiclone in elderly patients with primary insomnia. Sleep 2005;28:720–7. 16477959 10.1093/sleep/28.6.720

[18] Hajak G Rodenbeck A Voderholzer U Doxepin in the treatment of primary insomnia: a placebo-controlled, double-blind, polysomnographic study. J Clin Psychiatry 2001;62:453–63. 11465523 10.4088/jcp.v62n0609

[19] Guo J Huang W Tang CY Effect of acupuncture on sleep quality and hyperarousal state in patients with primary insomnia: study protocol for a randomised controlled trial. BMJ Open 2016;6:e009594. 10.1136/bmjopen-2015-009594 PMC4785323 26956161

[20] Passos GS Poyares D Santana MG Effect of acute physical exercise on patients with chronic primary insomnia. J Clin Sleep Med 2010;6:270–5. 20572421 PMC2883039

[21] Yin X Gou M Xu J Efficacy and safety of acupuncture treatment on primary insomnia: a randomized controlled trial. Sleep Med 2017;37:193–200. 28899535 10.1016/j.sleep.2017.02.012

[22] Tsutsui S Zolipidem Study Group. A double-blind comparative study of zolpidem versus zopiclone in the treatment of chronic primary insomnia. J Int Med Res 2001;29:163–77. 11471853 10.1177/147323000102900303

[23] Huang YS Hsu SC Liu SI A double-blind, randomized, comparative study to evaluate the efficacy and safety of zaleplon versus zolpidem in shortening sleep latency in primary insomnia. Chang Gung Med J 2011;34:50–6. 21392474

[24] Edinger JD Olsen MK Stechuchak KM Cognitive behavioral therapy for patients with primary insomnia or insomnia associated predominantly with mixed psychiatric disorders: a randomized clinical trial. Sleep 2009;32:499–510. 19413144 10.1093/sleep/32.4.499 PMC2663864

[25] Edinger JD Wohlgemuth WK Radtke RA Cognitive behavioral therapy for treatment of chronic primary insomnia: a randomized controlled trial. JAMA 2001;285:1856–64. 11308399 10.1001/jama.285.14.1856

[26] Sivertsen B Omvik S Pallesen S Cognitive behavioral therapy vs zopiclone for treatment of chronic primary insomnia in older adults: a randomized controlled trial. JAMA 2006;295:2851–8. 16804151 10.1001/jama.295.24.2851

[27] Zhang HJ Wang HB Lu F A randomized controlled study of cognitive behavioral therapy combined with drugs in the treatment of primary insomnia. Chin J New Drugs Clin Remedies 2010;29:426–9.

[28] Zhang HJ Yao Y Zhang JW A comparative study of cognitive-behavioral therapy and combination therapy for primary insomnia. Chin J Pract Nerv Dis 2010;13:6–9.

[29] Sutton AJ Duval SJ Tweedie RL Empirical assessment of effect of publication bias on meta-analyses. BMJ 2000;320:1574–7. 10845965 10.1136/bmj.320.7249.1574 PMC27401

[30] Egger M Davey Smith G Schneider M Bias in meta-analysis detected by a simple, graphical test. BMJ 1997;315:629–34. 9310563 10.1136/bmj.315.7109.629 PMC2127453

